# Vascular access for lipid apheresis: a challenge in young children with homozygous familial hypercholesterolemia

**DOI:** 10.1186/s12887-022-03192-7

**Published:** 2022-03-12

**Authors:** Julia Lischka, Klaus Arbeiter, Charlotte de Gier, Andrea Willfort-Ehringer, Nina-Katharina Walleczek, Renata Gellai, Michael Boehm, Albert Wiegman, Susanne Greber-Platzer

**Affiliations:** 1 Clinical Division of Pediatric Pulmonology, Allergology and Endocrinology, Department for Pediatrics and Adolescent Medicine, Comprehensive Center for Pediatrics, Medical University of Vienna, Waehringer Guertel 18-20, 1090 Vienna, Austria; 2 Division of Pediatric Nephrology and Gastroenterology, Department for Pediatrics and Adolescent Medicine, Comprehensive Center for Pediatrics, Medical University of Vienna, Waehringer Guertel 18-20, 1090 Vienna, Austria; 3 Division of Angiology, Department of Medicine II, Medical University of Vienna, Waehringer Guertel 18-20, 1090 Vienna, Austria; 4 Department of Pediatric Medicine, Amsterdam University Medical Centers, Location AMC, Meibergdreef 9, 1105 AZ Amsterdam, the Netherlands

**Keywords:** Vascular access, Children, Pediatric, LDL-cholesterol, Lipid apheresis, Arteriovenous fistula

## Abstract

**Background:**

Homozygous familial hypercholesterolemia (hoFH) is a rare genetic disorder leading to extremely increased LDL-cholesterol (LDL-C), resulting in high cardiovascular risk in early childhood. Lipid apheresis (LA) is an effective treatment and should be started as early as possible to prevent premature cardiovascular events. As peripheral punctures in children can be challenging due to small vessels and anxiety, this study aimed to evaluate feasibility and safety of central venous catheters (CVCs) as vascular access for LA in young children with hoFH.

**Methods:**

Retrospective analysis (2016-2019) on four children with hoFH aged 3-5 years, performing weekly or biweekly LA with a CVC.

**Results:**

LDL-C decreased by> 60%. In three children, the use of a permanent CVC for 698, 595, and 411 days, respectively, avoided difficult peripheral access, without the occurrence of occlusion or thrombosis. Unfortunately, one child had recurrent CVC-related infections and needed an arteriovenous fistula from the age of 5. Although the mean dwell time per catheter was 212 days, there were, as expected, severe side effects of early catheter infections with sepsis and accidental self-removal. Starting LA at an early age improved or stabilized carotid intima-media thickness (IMT) in three children. However, IMT did increase in one child caused by intolerance to peripheral punctures and LA interruption.

**Conclusions:**

Permanent CVCs are a viable temporary access choice for LA in young children with hoFH until peripheral venipuncture is practicable. The risk of CVC-related infections needs to be taken into account.

**Supplementary Information:**

The online version contains supplementary material available at 10.1186/s12887-022-03192-7.

## Background

Homozygous familial hypercholesterolemia (hoFH) is a rare genetic disorder with an estimated prevalence of 1:160,000 – 300,000 [1]. HoFH leads to a significant increase in LDL cholesterol (LDL-C) from birth onwards (above 500 mg/dL), resulting in high cardiovascular risk in early childhood. Hence, the earliest initiation of lipid-lowering treatment is required to prevent premature cardiovascular disease [1–4].

Fat-modified diets and lipid-lowering medications are hoFH treatment strategies, with minimal success [1, 2, 5]. Therefore, lipid apheresis (LA) plus statins- ezetimibe combination therapy has become standard practice to reduce LDL-C levels [6]. Expert consensus recommends reducing LDL-C levels by 50% in heterozygous FH (heFH) children, with a target LDL-C < 130 mg/dL from 10 years onwards [1] or < 135 mg/dL in pediatric hoFH [2]. There are no clear recommendations for younger children [1, 2].

Children suffering from hoFH are at very high risk for cardiovascular events, with reported cases of 3- to 4-year-olds manifesting angina pectoris and even fatal myocardial infarctions [7–10]. As untreated hoFH patients have a life expectancy of less than 30 years [2], guidelines recommend starting LA as early as possible [2, 10], preferably at age 2 [1], to decrease the risk of cardiovascular diseases and prolong life expectancy. Atherosclerotic cardiovascular disease is already present in asymptomatic children. Early signs include coronary ostial occlusion and aortic valve abnormalities, mainly aortic stenosis [2, 3]. Cutaneous or tendon xanthoma and arcus lipoides are typical features in the physical examination [1, 2].

LA is a highly effective method to reduce LDL-C with an expected mean reduction of up to 80% after a single treatment [11]. Promising new therapeutic approaches like the microsomal triglyceride transfer protein (MTP) inhibitor (Lomitapide) and the angiopoietin-like protein 3 (ANGPTL3)-targeting antibody (Evinacumab) can reduce LDL by about 50% in hoFH, but are not yet approved for children and adolescents. PCSK9 inhibitors reduce the degradation of the LDL receptor and are therefore not or only marginally effective in hoFH patients with pathological mutations in both alleles of the LDL receptor [1, 5, 12]. Venous access is needed for weekly or biweekly LA procedures [13]. However, repeated peripheral punctures to achieve an adequate blood flow is often challenging in young children due to small peripheral vessels and severe anxiety [6, 14, 15]. Permanent central venous catheters (CVCs) or arteriovenous fistulas (AVF) are possible alternatives and usually optimize treatment. Moreover, CVCs allow children to move their arms and body, thus being better tolerated [1, 2, 5, 15].

While vascular access options for hemodialysis have been extensively reviewed [14, 16, 17], there is limited evidence on surgical venous access in LA [18–20]. Only a few studies reported on hypercholesterolemic patients [21], one case study [22], and one recently published systematic review [15] investigated LA in children with hoFH specifically. However, research on venous access techniques in pediatric patients is still lacking, making an evidence-based approach difficult.

The aim of this study was to evaluate feasibility and safety of central venous catheters as a possible venous access for regular LA in young children with hoFH.

## Methods

The study includes young children from the Outpatient Clinic for Pediatric Obesity and Dyslipidemia of the Department of Pediatrics and Adolescent Medicine at the Medical University of Vienna, who underwent LA between October 2016 and November 2019. Inclusion criteria were the presence of genetically confirmed homozygous familial hypercholesterolemia requiring regular LA and aged between 1 to 6 years.

LA, which selectively removes apolipoprotein B (ApoB) 100-containing lipoproteins from the patients’ blood via extracorporeal filtration, was performed weekly or biweekly. A Therasorb column by Miltenyi Biotec was used with a plasma Adasorb filled with 5% albumin and a volume of 100 ml. LA volume settings were the same for peripheral and central venous access. Systemic anticoagulation consisted of bolus administration of citrate and heparin, followed by a continuous citrate/heparin infusion.

Permanent (tunneled) CVCs (Permcath, Quinton Instrument Co., Seattle, USA or Broviac, BARD Access Systems, Salt Lake City, USA) were surgically implanted in the subclavian or jugular veins. The CVC lines were filled with an alteplase solution in interval periods.

All four study participants underwent physical examinations, and medical history, clinical and laboratory data were collected.

Lipid levels, physical and additional clinical examinations like electrocardiogram (ECG), echocardiography, supra-aortic intima-media thickness (IMT), and coronary CT / coronary catheter were analyzed at baseline, during LA, and at least after 3 years (up to 1138 days) of LA.

IMT was measured by carotid ultrasound at the Division of Angiology, Department of Internal Medicine at the Medical University of Vienna. Mean IMT was calculated out of 12 consecutive measurements per each side. The clinical assessment referred to age- and sex-specific reference values based on internal data (unpublished).

CVC-related complications were classified into six subgroups according to the literature: mechanical (dislocation, migration, rupture, self-removal by accident) [23–25]; malfunction/occlusion (except thrombosis) [23, 24, 26]; thrombotic (thrombosis, embolism, deep vein thrombosis) [23, 24]; infectious (local vs. systemic; CRBSI: catheter-related bloodstream infection) [23, 24, 26]; complications associated with implantation (pneumo-, hydro-, hemothorax, arterial puncture, cardiac tamponade, arrhythmia, bleeding, air embolism, vessel perforation, hematoma) [23, 25]; and not otherwise classified complications e.g., loss of central venous patency after multiple CVC insertions [27, 28].

### Statistics

Due to the small sample size, descriptive statistics were used to analyze the data. Categorical data were analyzed using absolute frequencies and relative frequencies.

All statistical analyses were performed using Microsoft Excel 2010 for Windows 7 (Microsoft Corp., Redmond, WA, USA).

### Ethics

The study protocol was approved by the ethics committee of the Medical University of Vienna (No. 1638/2019) and conforms to the Helsinki declaration guidelines.

## Results

### Patient characteristics

LA was started at the age of 3 to 5 years in four children with severe hoFH (molecularly proven and LDL-c > 500 mg/dL after diet and lipid-lowering medication). Statins (simvastatin or atorvastatin, 2 mg/kg, max. 40 mg/d), ezetimibe (10 mg), and colesevelam (625 mg 2x/day) were administered orally. Patient 1 refused colesevelam because of abdominal side effects.

### Vascular access

**Fig. 1:**
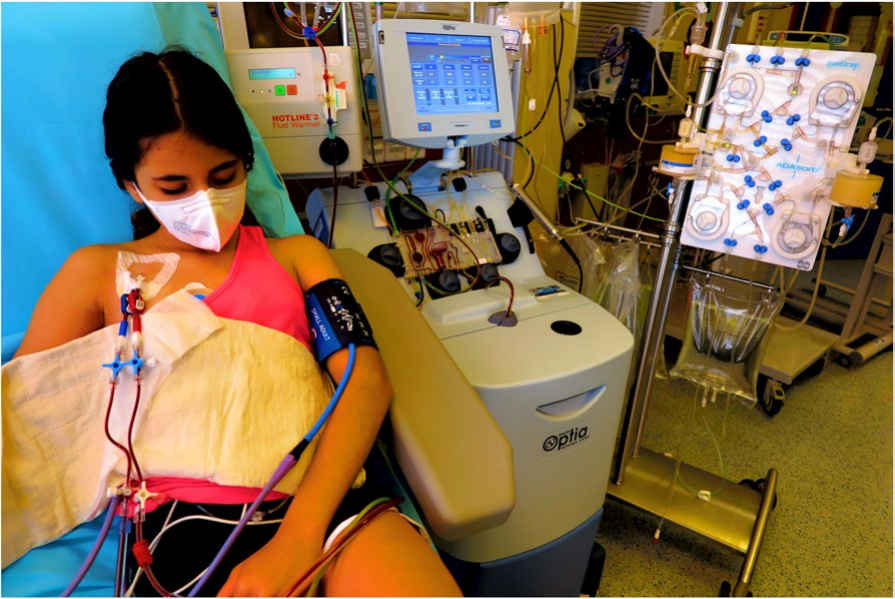
Patient 1 during lipid apheresis. The central venous catheter is inserted in the Vena jugularis interna dextra

Initially, the permanent CVCs were surgically implanted in four children with hoFH (three girls, one boy) aged 3 to 5 (Fig. 1). All patients started with biweekly LA, later intensified to weekly intervals for three of them. Patient 4 had several CVC complications and therefore received an AVF 8 months later, at the age of 5 years. The venous situation and tolerance to peripheral venous punctures progressively improved in the three female patients; therefore, CVCs could be removed at the age of 6 years in one girl and 7 years in the other two. (Table 1).

**Table 1:** Patient characteristics

Patient	Sex (w/m)	Genetic mutation	Age at diagnosis (years)	Period of CVC use (age in years)	Cumulative months of CVC use	Age at follow-up (years)	Venous access at follow-up
1	w	LDLR homozygous	3	5 to 7	24	8	peripheral
2	w	LDLR homozygous	2	4 to 6	23	7	peripheral
3	w	LDLR homozygous	2	5 to 7	30	8	peripheral
4	m	LDLR homozygous	1	3 to 5	8	7	AVF

### Lipid apheresis

Between October 2016 and November 2019, the four patients had a total of 106, 104, 75, and 94 LA sessions, respectively. In Patient 1, starting LA aged 5, the critical peripheral venous situation required a CVC implantation due to high blood flow. Infections and accidental self-removal of the CVCs occurred five times, and in between, several unsuccessful venous punctures were attempted, which were painful and stressful for the child, despite extensive psychological support. Nine LA sessions were missed, and four were interrupted. The implantation of a new CVC stabilized LDL-C values.

All four children needed iron supplementation throughout LA, indicating chronic iron deficiency anemia. Additionally, liposoluble vitamins (vitamins A and D, substituted with Oleovit D3 or multivitamin supplement Advit) had to be supplemented. Other side effects related to LA like hypotension and hypocalcemia were not observed. During LA additional intravenous fluid infusion was never necessary.

### Treatment outcome

Mean baseline LDL-C levels with a maximum dose of lipid-lowering medication before the start of LA ranged from 535 to 742 mg/dL. With LA, mean LDL-C levels decreased to 244 up to 411 mg/dL pre-LA, and 76 up to 154 mg/dL post-LA (Table 2). Thus, LA reduced LDL-C levels by more than 60% in all four children. As a positive side effect, LA also decreased Lipoprotein (a) by over 60%, if elevated (Supplementary Table 1).

**Table 2:** Lipid apheresis: Lipid levels at baseline and after 3 years of lipid apheresis

Patient	LDL-C [mg/dL] on max. Oral medication	Steady state Pre-LA LDL-C [mg/dL]	Steady state Post-LA LDL-C [mg/dL]
1	688.8^a^	411.2 (288.8-564.6)	153.5 (113.8-227.8)
2	535.4 (534.8-536.0)	337.2 (265.0-443.0)	104.7 (84.8-151.6)
3	597.1 (521.2-732.0)	244.2^b^ (223.0-287.4)	81.0^b^ (67.4-99.0)
4	742.0^a^	251.7 (205.0-297.4)	76.4 (57.2-94.8)

Values at baseline are on maximum oral medication with statins and ezetimibe in all patients and colesevelam in Patient 2. Lipid values were measured before and after weekly/biweekly LA. Values are displayed as means, minimum and maximum over a period of 3 months. ^a^only one measurement available. ^b^biweekly

In three children undergoing weekly LA procedures, the pre-LA LDL-C remained high 1 week after LA, with values between 252 to 411 mg/d, whereas one girl continued biweekly sessions, with pre-LA LDL-C values of around 244 mg/dL 2 weeks after LA (Table 2).

After 3 years of LA treatment, skin xanthomas decreased or disappeared in all patients. In detail, Patient 1 showed a remission of the xanthoma on the elbows, knees, and upper legs. In Patient 2, the xanthoma on both Achilles tendons, knees, and dorsum of both feet nearly disappeared except for small residual on the left knee and right Achilles tendon. In Patient 3, the xanthoma on hands, knees, and dorsum of both feet decreased, and the arcus lipoides diminished. Patient 4 showed complete remission of the xanthoma on both Achilles tendons, right knee, and left foot dorsum.

Pre-treatment echocardiography revealed mild aortic insufficiency in two children, while another child had mild aortic insufficiency at 3-year follow-up. Three children showed increased age- and gender-specific baseline carotid IMT levels, which remained unchanged, decreased, or normalized after 3 years of LA treatment (Supplementary Table 2). Patient 1 developed a new vascular plaque formation in the right internal carotid artery, without hemodynamic significance. Before starting LA, a coronary CT angiography performed on all patients revealed normal coronary arteries without plaque formation or stenosis.

### Complications associated with vascular access

Complications associated with CVCs are listed in Table 3. Infections and mechanical complications occurred most frequently. All four patients suffered from a bacterial infection progressing to bacterial sepsis in three instances (Table 3): *Staphylococcus aureus* in five cases and *Plesiomonas shigelloides* in one.

**Table 3:** Central venous catheter complications

Complications	Number total	Number of patients with at least 1 event
**Infections**	**9**	**4**
-) Systemic infection: Sepsis/SIRS	3	2
-) Systemic infection: CRBSI	4	2
-) Local CVC-infection	2	1
**Mechanical**	**9**	**3**
-) Self-removal by accident	6	3
-) Dislocation	3	2
**Malfunction: occlusion**	**0**	**0**
**Thrombosis**	**0**	**0**
**Complications associated with implantation**	**0**	**0**
**Other:** Pneumo-Mediastinum/Pericard	**1**	**1**

Different subgroups defined as CVC related complications. Each patient suffered from at least one infection, in 3 cases sepsis occurred. The most frequent complication was accidental self-removal of the catheter. *CRBSI* Catheter-Related Blood Stream Infection

Patient 1 was hospitalized twice for 13 and 15 days, respectively, due to catheter-related sepsis caused by *Staphylococcus aureus*, and each time a new CVC had to be implanted. The family excluded the option to place an AVF.

Patient 2, who presented with catheter related complications twice, had an CRBSI event that resolved without further issues.

Patient 3 experienced one severe complication shortly after the first CVC implantation and had to be hospitalized with systemic inflammatory response syndrome that later induced staphylococcal scalded skin syndrome.

Patient 4 had multiple catheter-related complications in the first year of LA: four CVC dislocations, leading to catheter loss twice, and three episodes of CRBSI requiring hospitalization. Therefore, he received an AVF 8 months later (Table 4).

**Table 4:** Site of venous access, dwell time and reason for removal of central venous catheter per patient in chronological order

Patient	Site of venous access	Dwell time in days	Reason for removal
**1**	V. jug. sin.	19	Sepsis
	V. subcl. sin.	411	Sepsis, Self-removal by accident
	V. jug. sin.	51	Self-removal by accident
	V. subcl. dex.	66	Self-removal by accident
	V. subcl. sin.	183	Dislocation, local infection
	Peripheral access	at the age of 7 years	
**2**	V. subcl. sin.	698	Self-removal by accident
	Peripheral access	at the age of 6 years	
**3**	V. jug. sin.	14	Sepsis
	V. subcl. sin.	595	Elective removal
	V. subcl. dex.	280	Elective removal
	Peripheral access	at the age of 7 years	
**4**	V. jug. sin.	71	Self-removal by accident
	V. subcl. sin.	55	CRBSI, Self-removal by accident
	V. jug. dext.	102	CRBSI
	AVF	at the age of 5 years	
Mean dwell time per catheter		212	

Two girls, Patients 2 and 3 tolerated the CVCs with only few complications and maximum dwell times of 698, and 595 days, respectively. Patients 1 and 4 suffered from repeated infections and dislocations, therefore, Patient 4 received an AVF after 8 months of LA. *CRBSI* Catheter-Related Blood Stream Infection

Mechanical complications such as accidental self-removal and dislocation of the CVCs occurred in three children. Patient 1 needed five CVCs in total (Table 4).

In two girls, Patients 2 and 3, CVCs were stable and could be used for 595 days (19 months) and 698 days (23 months), respectively (Table 4). After accidental self-removal in Patient 2 at the age of 6, the switch to peripheral punctures succeeded, thanks to the emotional support given by the child’s mother. Peripheral puncture attempts in Patient 3 at the age of 7 were less successful due to anxiety, stress, and psychological pressure, requiring implantation of a new CVC 1.5 months later.

Aged 6, Patient 1 referred thoracic pain and dyspnea suddenly after a ball shot to the upper thorax during school sports. Small pneumomediastinum and pneumopericardium were diagnosed, and the girl was hospitalized for observation. The CVC was still intact, and the air was rapidly absorbed without further complications.

No other catheter-related complications like occlusion, venous thrombosis, or perioperative problems were observed (Table 3). Ultrasound measurement always showed normal central veins anatomy and blood flow.

In total, there were 12 implantations/re-implantations of CVCs in four young children with hoFH treated with LA over 3 years. Patency of CVCs was most difficult to maintain within the first months. Six out of twelve CVCs were still in place after 3 months. Predominant reasons for early CVC loss were infection or accidental self-removal. Long-term patency rates were otherwise stable.

## Discussion

This observational, retrospective study is the first evaluation of permanent CVCs as venous access for LA in young children with hoFH. Twelve CVC implantations/re-implantations were necessary for four young children aged 3-7 years. As one boy suffered several CVC-related infections within the first year, the AVF was preferred. CVCs were used on three children until peripheral punctures were tolerated, at age 6 to 7, and avoided AVF placement.

In this study, children aged 3 to 5 started LA with CVCs as venous access. In a publication from 2017, LA initiation depended on the patient’s tolerance of peripheral venous puncture; therefore the procedure was never performed before the age of 6 years [29]. A systematic review by Luirink et al. recently highlighted the importance of starting LA as early as possible. Of the 123 analyzed patients with hoFH, 17% sustained a cardiovascular event before accessing LA treatment aged between 5 to 15. The average age at the start of LA was 9.3 years, with only 12% of patients undergoing the procedure before age 5 [15]. De facto, performing LA on young children is challenging because of small peripheral vessels raising technical problems related to venous puncture, low blood flow, and low blood volume [15, 30]. The clinical benefit of LA in hoFH patients refers to the positive effect on life expectancy [31–33]. In heFH patients under LA treatment, the incidence of coronary events was reduced by 72% compared to the treatment with lipid-lowering drugs only [32].

Anxiety and emotional distress can affect patients’ compliance with LA treatment. After several peripheral punctures attempts to allow a 7-year-old girl (Patient 3) more sporting activities, especially in the summer, such as swimming, her severe anxiety led to the decision to opt for a CVC. Nine months later, with intensive psychological support, and a better understanding of the illness, the child tolerated peripheral punctures and the CVC was electively removed. Psychological factors appear to be a concern even with the medical and nursing staff expertise, extensive psychological support, and local anesthesia.

In this small study cohort, infections and accidental self-removal were the most frequent complications. In summary, seven systemic infections (3 sepsis, 4 CRBSI) occurred in four children with hoFH. The cumulative CRBSI rate was 2.8 per 1000 CVC days. Significantly more than previously published by Pinon et al., who reported a rate of 0.46 per 1000 CVC days in a pediatric cohort of oncological patients aged up to 20 (mean age 7.3 years) receiving long-term medication [25]. Former studies confirmed that young children are at increased risk of CVC infections [23, 26] and that long-term use of indwelling CVCs is a risk factor for CRBSI [34].

We observed frequent systemic and local infections in our cohort shortly after CVC implantation, which required central catheter replacement. Six out of twelve CVCs were still in situ after 3 months, resulting in a cumulative short-term duration rate of 50%. Cesaro et al. reported that young age (under 5 years) is a predictor for premature CVC removal, particularly in the first 2 months after implantation [26].

Half of the CVCs were lost by accidental self-removal and needed catheter reimplantation. Estimates of dislocation rates in the literature are 31% of all complications associated with CVCs in children [25]. Young age is a known risk factor for dislocations [23, 25, 35] which mainly occur early after implantation and may also be caused by incomplete cauterization of the cuff leading to poor adhesion [25].

Despite CVCs being the leading cause of thrombotic events in children, no catheter occlusion or thrombosis were observed in this study [36, 37]. Most research on CVCs in pediatric cohorts assessed children with cancer; hence the prothrombotic state associated with hemato-oncological diseases could explain the higher rate of thrombosis in the literature [24–26]. However, LA influences coagulation and perhaps prevents thrombosis [38].

A severe long-term effect of repeated CVC insertions is vascular occlusion [26, 27]. Regular doppler monitoring of supra-aortic blood flow revealed the absence of occlusions in the four children with hoFH.

Several studies proved that LA treatment promotes xanthoma regression, plaque stabilization, and even vascular regeneration [2, 15, 39]. We observed regression of xanthoma and no increase in supra-aortic IMT in three children. Only one girl (Patient 1) showed a slight IMT increase and developed a vascular plaque formation in the left internal carotid artery during the 3 years of LA, potentially resulting from frequent treatment interruptions due to the patients’ non-compliance, catheter removal, or infections. Notably, after accidental CVC self-removal occurred in the second year of LA, only nine out of 21 LA sessions could be completed due to failure or intolerance to peripheral venous access.

While early-onset LA prevents premature cardiovascular events, obtaining venous access in young children may be problematic. There are three options for venous access, including peripheral venous punctures, forearm arteriovenous fistula (AVF), or permanent central venous catheters. Small-caliber veins, stress, and anxiety exclude peripheral venous punctures in younger children. The AVF procedure requires the vascular specialist to connect very small vessels and minimize the risk associated with the AV fistula, while the patient should be able to withstand the punctures during dialysis sessions [28, 40]. Moreover, AVFs in FH patients seem to be associated with a higher risk of vascular diseases [21, 41–43]. Central venous catheters avoid repeated treatments involving needles insertion and allow some movements during the LA procedure. On the other hand, the high risk of infections, dislocations, and other complications indicate a well-considered use, as described for hoFH children needing regular LA.

A limitation of this study may be the small sample size, although epidemiological data estimate the frequency of hoFH to be 1:160,000 – 1:300,000, categorizing the disease as rare [1]. Therefore, it is relevant and valuable to describe pros and cons of CVCs used in young hoFH children to enable LA early on, as the only effective therapy. The new therapeutic options like lomitapide and ANGPTL3-inhibitors appear to be successful in treating adult hoFH patients, but are currently of limited use in pediatric patients, since side effects and missing drug approval have to be considered. In the future, more potent lipid lowering drugs approved for children will help to reduce or even stop invasive procedures like LA [5].

## Conclusions

We can conclude that although central venous catheters are an option to start LA in young children with severe hoFH, this type of access needs careful management to avoid infections and dislocations and should only be used when urgency overrules contraindications. Moreover, it is essential to describe the pros and cons of CVCs in the youngest hoFH population to enable LA at an early stage as the only effective therapy. Delaying the start of LA exposes patients to premature cardiovascular events already in early childhood. Ultimately, AVFs can be an alternative to CVCs; however, the possibility to perform venipuncture earlier in life, subject to the patient’s cooperation and vessel diameter, is preferable.

More data on venous access options for LA in early childhood is necessary to adequately assess the advantages and disadvantages of different approaches and guide clinical decision-making for the individual patient.

## Supplementary Information

**Additional file 1: Supplementary Table 1.** Lipid apheresis: Lipid levels at baseline and after 3 years of lipid apheresis. HDL-C, total cholesterol and lipoprotein (a) values are displayed. Lipid values were measured before and after weekly/biweekly LA. Values are displayed as means, minimum and maximum over a period of 3 months.

**Additional file 2: Supplementary Table 2.** Patient characteristics: IMT measurements. All IMT measurements are shown for each patient individually. Values are displayed for the right / left common carotid artery, respectively.

## Data Availability

All data generated or analyzed during this study are included in this published article (and its supplementary information files).

## Abbreviations

CVC: Central venous catheter
LA: Lipid apheresis
AVF: Arteriovenous fistula
LDL-C: Low-density lipoprotein cholesterol
hoFH: Homozygous familial hypercholesterolemia
heFH: Heterozygous familial hypercholesterolemia
IMT: Carotid intima-media thickness
ApoB: Apolipoprotein B
ECG: Electrocardiogram
CRBSI: Catheter-related bloodstream infection
SIRS: Systemic inflammatory response syndrome
