# Supplementary material for: Vascular access for lipid apheresis: a challenge in young children with homozygous familial hypercholesterolemia
Source: BMC Pediatr. 2022 Mar 12;22:131. doi: 10.1186/s12887-022-03192-7 (PMC8917672; doi:10.1186/s12887-022-03192-7)
Supplement: Supplementary file 1 — Additional file 1: Supplementary Table 1. Lipid apheresis: Lipid levels at baseline and after 3 years of lipid apheresis. HDL-C, total cholesterol and lipoprotein (a) values are displayed. Lipid values were measured before and after weekly/biweekly LA. Values are displayed as means, minimum and maximum over a period of 3 months. [file 12887_2022_3192_MOESM1_ESM.pdf]

Supplementary Table 1 Lipid apheresis: Lipid levels at baseline and after 3 years of lipid apheresis.

Steady state Pre-LA			
**Patient**	**HDL-C [mg/dl]**	**Total cholesterol [mg/dl]**	**Lipoprotein (a) [nmol/l]**
1	28.62 (25.00-33.00)	470.15 (341.00-626.00)	12.00 (11.00-13.00)
2	49.31 (43.00-53.00)	412.62 (324.00-509.00)	23.00 (11.00-31.00)
3[a]	46.33 (40.00-51.00)	332.67 (298.00-372.00)	194.33 (165.00-219.00)
4	52.38 (48.00-57.00)	317.17 (273.00-359.00)	180.67 (147.00-208.00)

Steady state Post-LA			
**Patient**	**HDL-C [mg/dl]**	**Total cholesterol [mg/dl]**	**Lipoprotein (a) [nmol/l]**
1	17.08 (13.00-20.00)	190.31 (143.00-266.00)	7.00 (7.00-7.00)
2	30.62 (28.00-35.00)	146.00 (123.00-202.00)	8.33 (7.00-10.00)
3[a]	29.50 (26.00-34.00)	123.83 (114.00-145.00)	55.67 (44.00-65.00)
4	33.00 (29.00-37.00)	117.69 (94.00-140.00)	60.33 (48.00-76.00)

Lipid values were measured before and after weekly/biweekly LA. Values are displayed as means, minimum and maximum over a period of 3 months. [a]biweekly
