# Supplementary material for: Vascular access for lipid apheresis: a challenge in young children with homozygous familial hypercholesterolemia
Source: BMC Pediatr. 2022 Mar 12;22:131. doi: 10.1186/s12887-022-03192-7 (PMC8917672; doi:10.1186/s12887-022-03192-7)
Supplement: Supplementary file 2 — Additional file 2: Supplementary Table 2. Patient characteristics: IMT measurements. All IMT measurements are shown for each patient individually. Values are displayed for the right / left common carotid artery, respectively. [file 12887_2022_3192_MOESM2_ESM.pdf]

Supplementary Table 2 Patient characteristics: IMT measurements

Patient 1

Date	IMT [mm]	Plaque
24.08.2016	0.43/0.43*	<25% A. carot. ext. sin.
04.05.2017	0.42/0.34*	<20% A. carot. int. dex./<20% A. carot. ext. sin.
05.04.2018	0.34/0.34*	<20% A. carot. int. dex./<20% A. carot. ext. sin.
12.12.2018	0.42/0.36*	<20% A. carot. ext. sin./<20% Art. carot. comm.

Patient 2

Date	IMT [mm]	Plaque
24.09.2015	0.33/0.30*	no plaque
07.06.2017	0.18/0.13	no plaque
12.04.2018	0.27/0.32*	no plaque
14.11.2018	0.29/0.30	no plaque
21.08.2019	0.30/0.30	no plaque

Patient 3

Date	IMT [mm]	Plaque
25.05.2016	0.20/0.23	no plaque
19.05.2017	0.24/0.23	no plaque
25.04.2018	0.19/0.19	no plaque
16.01.2019	0.11/0.13	no plaque

Patient 4

Date	IMT [mm]	Plaque
13.01.2016	0.32/0.33*	no plaque
19.05.2017	0.34/0.31*	no plaque
26.01.2018	0.33/0.32*	no plaque
10.04.2018	0.29/0.30	no plaque
06.02.2019	0.14/0.20	no plaque

Values are displayed for the right / left common carotid artery, respectively.
*increased IMT
